# Automatic Classification of Gait Impairments Using a Markerless 2D Video-Based System

**DOI:** 10.3390/s18092743

**Published:** 2018-08-21

**Authors:** Tanmay T. Verlekar, Luís D. Soares, Paulo L. Correia

**Affiliations:** 1 Instituto de Telecomunicações, Instituto Superior Técnico, 1049-001 Lisbon, Portugal; plc@lx.it.pt; 2 Instituto de Telecomunicações, Instituto Universitário de Lisboa (ISCTE-IUL), 1649-026 Lisbon, Portugal; lds@lx.it.pt

**Keywords:** gait analysis, biomechanical gait features, impaired gait classification

## Abstract

Systemic disorders affecting an individual can cause gait impairments. Successful acquisition and evaluation of features representing such impairments make it possible to estimate the severity of those disorders, which is important information for monitoring patients’ health evolution. However, current state-of-the-art systems perform the acquisition and evaluation of these features in specially equipped laboratories, typically limiting the periodicity of evaluations. With the objective of making health monitoring easier and more accessible, this paper presents a system that performs automatic detection and classification of gait impairments, based on the acquisition and evaluation of biomechanical gait features using a single 2D video camera. The system relies on two different types of features to perform classification: (i) feet-related features, such as step length, step length symmetry, fraction of foot flat during stance phase, normalized step count, speed; and (ii) body-related features, such as the amount of movement while walking, center of gravity shifts and torso orientation. The proposed system uses a support vector machine to decide whether the observed gait is normal or if it belongs to one of three different impaired gait groups. Results show that the proposed system outperforms existing markerless 2D video-based systems, with a classification accuracy of 98.8%.

## 1. Introduction

Gait can be defined as a coordinated, cyclic combination of movements that results in human locomotion. Being a highly cognitive task, the manner of walking is unique to an individual [1]. However, it can be altered because of physical injuries and systemic disorders. These injuries/disorders can affect human locomotion and posture, typically resulting in reduced walking speed and step length [2]. Gait of an individual under such circumstances can be considered as impaired. By analyzing biomechanical features derived from gait, such as speed, cadence, step length, stance time, or swing time, it is possible to infer whether the observed gait is impaired, and in some cases even distinguish between different disorders that cause gait impairments, and their severity [3]. The same set of biomechanical gait features can also be used to predict fall risks in elderly populations [4] or head impacts in athletes [5].

Nowadays, the acquisition and clinical evaluation of biomechanical gait features is performed in dedicated laboratories, using a sophisticated equipment setup and with the help of specialized personnel, resulting in an expensive and time-consuming task [6]. The goal of this paper is to present a novel system that performs automatic detection and classification of gait impairments, based on the acquisition and evaluation of biomechanical gait features, using a single 2D video camera, thus making its operation possible in daily life settings, such as in clinics or even at home.

### 1.1. State-of-the-Art

Biomechanical features for the evaluation of an individual’s gait can be acquired using several types of systems. Based on the acquisition process, they can be classified into sensor-based or vision-based systems [7]. Sensor-based systems use devices such as force sensitive resistors [8], pressure mats [9], accelerometers [10] and inertial measurement units (IMUs) [11], to acquire signals representing human motion. These sensors, which can be expensive or inaccessible to most individuals, can be setup up on the floor or attached to the body of the individual. The signals thus obtained can be processed to estimate biomechanical gait features such as velocity, cadence, step length and step time, which are effective in evaluating the individual’s gait [10,11]. Setting up the selected sensors can be complex, in some cases having to be done by clinical professionals, as is often the case with body worn sensors. The processing of the resulting signals, to extract biomechanical features and perform their analysis, is also done by trained professionals.

Vision-based systems rely on the use of cameras to capture image sequences, from which biomechanical gait features can be extracted. Depending on the way the captured information is represented and processed, these systems can be classified into [1]:

- Model-based systems;
- Appearance-based systems.

Model-based systems typically rely on the use of images obtained from multiple calibrated cameras, depth sensing cameras, or a combination of both, to model the gait of an individual [12]. The use of multiple calibrated cameras allows the systems to estimate features such as the height, distance between the feet [13], or the joint angles [14]. Their performance can be further improved by using fish-eye cameras and passive markers to highlight the key joint positions [15]. A widely used model-based system for clinical evaluations is the optoelectronic motion capture system. An example of such a system is presented in [6], with the configuration of six calibrated infra-red cameras, and forty-four passive markers attached to selected body positions, for characterizing an individual’s gait, while other configurations can be considered. Optoelectronic motion capture systems are considered as the gold standard for clinical evaluations, because of the accuracy of the features obtained. A drawback of this type of system is that it can only be operated in special laboratories due to the complex setup and the need for calibrations before use [7]. Thus, simpler setups have been proposed using depth sensing cameras to acquire the skeletal model of an individual, from which it is possible to estimate features [16] such as, joint positions [17], or motion history [18]. The accuracy of such systems is lower than that of the optoelectronic motion capture system, and their operation is typically limited to a range between 80 cm and 4 m.

On the other hand, appearance-based systems are typically markerless and rely on a single 2D camera, with the spatio-temporal information obtained from the captured video sequence being used to estimate biomechanical gait features. This system configuration was initially used for biometric recognition applications [19], performing well even under unconstrained conditions [20,21]. The biometric features used for recognition can be derived from representations such as the gait energy image (GEI). Such features have also been used to perform classification of gait disorders, for instance resulting from Parkinson’s disease, neuropathy, hemiplegia and diplegia [22], or to detect the amount of movement and movement broadness of an individual’s feet [23].

Different gait representations can be considered to better characterize an individual’s health. Several appearance-based systems extract biomechanical gait features, such as step length, leg angles, gait cycle time [24,25] cadence, speed, and stride length [26], or the fraction of the stance and swing phases during a gait cycle [27], using the body silhouettes computed from a 2D video sequence. The above features are distinctive enough to classify gait as normal or impaired. Appearance-based systems can also estimate posture instabilities using biomechanical features such as lean and ramp angles [28], axial ratio and change in velocity [29]. Other appearance-based systems distinguish between normal and wavering, faltering or falling gait using features such as homeomorphisms between 2D lattices of binary silhouettes [30].

Appearance-based systems based on 2D video do not have access to depth information, which can limit their accuracy when compared to some sensor or model-based systems. However, the major body articulations are clearly visible during a gait cycle, notably if video is captured from a lateral viewpoint, often designated as the canonical view [31]. In those conditions, the features obtained from a single 2D video camera contain enough information to characterize an individual’s gait, with the advantage that the system is much easier to install and operate in a daily life setting, when compared to model or sensor-based systems.

### 1.2. Motivation and Contribution

Most state-of-the-art markerless systems using a single 2D camera only perform a binary classification of gait as being either normal or impaired, since the classification of the type of gait impairment is a significantly more challenging task given the biomechanical gait features that are typically used. However, such classifications can provide a preliminary assessment of the type or the severity of a disorder. The automatic classification performed by the proposed system, also makes such preliminary assessments accessible to individuals in a daily life setting, where a constant presence of trained professionals is not possible.

Some systems, such as the one reported in [22], can distinguish between different types of gait disorders using biometric gait features derived from a GEI. However, their performance is poor when trying to distinguish gait disorders such as diplegia and Parkinson, as the resulting GEIs are very similar [22]. In such cases, the usage of additional biomechanical gait features, such as leg joint angles [22], or step length, foot flat ratio and speed, can lead to a significantly better performance. However, obtaining these features from a 2D video sequence can be challenging, with self-occlusions and the lack of depth information often leading to poor classification results. The self-occlusion problem can be especially difficult to handle for some gait impairments caused by disorders such as Parkinson’s disease, where the short strides cause the feet to remain occluded throughout the gait cycle, as discussed in Section 2.2.

This paper presents a novel markerless appearance-based system that acquires biomechanical gait features from a single 2D camera, able to describe an individual’s gait even under self-occlusions. These features include step length, foot flat ratio, speed, normalized step count, torso orientation, shift in center of gravity (COG) and the amount of movement while walking. Most state-of-the-art markerless 2D video based systems that compute step length, do not differentiate between the left and the right leg. Thus, the proposed system improves on the state-of-the-art by distinguishing the left and right step lengths, which allows identifying the limb(s) contributing to the impaired gait. Also, the proposed “amount of movement” feature helps to identify motion restricted limbs. Together, these features can be used to measure gait symmetry. The proposed system also computes a temporal feature, “foot flat ratio”, that estimates the fraction of time during which the foot is in complete contact with the ground during the stance phase. This feature is significantly more descriptive than the previously considered stance/swing phase during a gait cycle [23], and along with speed and normalized step count it allows the proposed system to more reliably detect deviations from normal gait. Also, novel body related features are proposed, such as torso orientation and shift in COG, which allow estimating posture instabilities, such as a hunchback. The proposed system can therefore detect gait impairments and, in a first stage, classify an individual as having gait impairments affecting the left, the right or both sides of the body.

The rest of the paper is organized as follows: Section 2 presents the proposed system and the acquired biomechanical gait features. Section 3 presents experimental results. Section 4 provides a discussion about the quality of the features obtained, and the classification accuracy of the proposed system using a support vector machine (SVM) classifier. Section 5 presents conclusions and suggests directions for future work.

## 2. Methods

**Figure 1:**
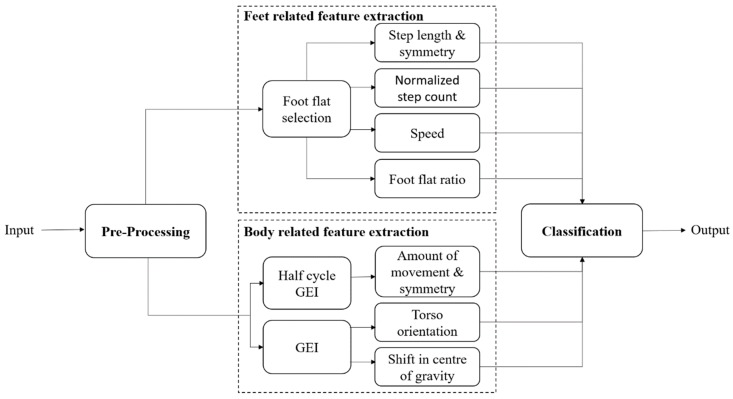
Proposed system architecture.

The architecture of the proposed system, which takes a 2D video as input, is presented in Figure 1. After an initial pre-processing step to extract binary silhouettes from the 2D video, the following two sets of biomechanical gait features are computed:

- Feet-related features—These features are obtained using the spatio-temporal information available from the individual’s feet area and include: (i) left and right step lengths, and step length symmetry; (ii) normalized step count; (iii) speed; and (iv) left and right foot flat ratios;
- Body-related features—These features are obtained using information from the entire body of an individual, thus reflecting posture, and include features such as: (i) amount of movement in the left and the right side of the body, movement symmetry; (ii) shift in the COG with respect to its center of support (COS); and (iii) torso orientation.

These features can then be used to decide whether the observed gait is impaired and, if it is, to classify gait impairments into different groups. The proposed system performs the classification using an SVM. Each of the main modules of the proposed architecture are detailed in the following sections.

### 2.1. Pre-Processing

The proposed system performs background subtraction [32] on the input 2D video, to obtain a sequence of binary silhouettes of the walking individual. The silhouettes are then normalized with respect to height, while maintaining their original aspect ratio. The normalization step, applied to each frame, makes the proposed system robust to scale changes, such as those resulting from a varying distance between an individual and the camera. The distance between the feet of the individual is then approximated as the width of a rectangular bounding box fitted onto the silhouettes [33]. Using the silhouettes and the distance between the feet, the proposed system can compute the desired biomechanical gait features.

### 2.2. Feet Related Feature Extraction

**Figure 2:**
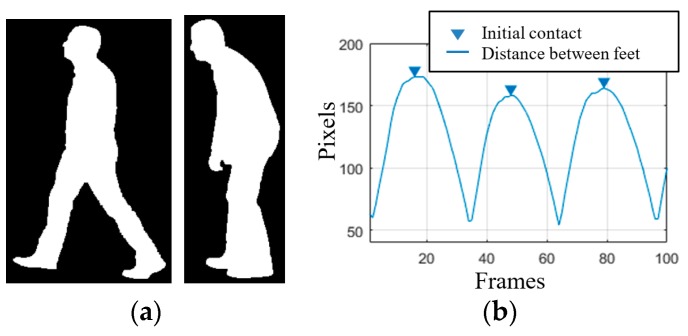
(**a**) Silhouettes belonging to a healthy individual (left) and an individual suffering from a systemic disorder (right); (**b**) plot representing the distance between feet along a gait sequence.

The gait of an individual consists of repetitions of a gait cycle. It begins and ends with an event called the “initial contact”, which occurs when the heel of the foot being observed first meets the ground. The distance covered between the initial contact of the observed (left or right) foot and the initial contact of the other foot is called step length. In a healthy individual, detecting the initial contact, and thus estimating the step length can be easy since the feet are spread wide apart while walking [24]. However, in the presence of certain impairments that affect gait, the strides can be extremely short, leading to self-occlusions (i.e., the part of the body closer to the camera occludes other parts of the body), as illustrated in the right side of Figure 2a. Under such conditions, identifying the exact instant of initial contact or estimating the step length using a silhouette can be difficult.

The proposed system tackles this problem by detecting the “foot flat” instants, defined as the part of the gait cycle during which the foot is in complete contact with the ground. A gait cycle includes two foot flat instants, one for each of the feet, occurring right after the initial contact. To minimize the effect of self-occlusions in the presence of very short step lengths, foot flat instances are detected by analyzing half of the gait cycle at a time, i.e., the span between two consecutive initial contact events (of opposite feet). However, since determining the exact instant of initial contact is difficult, the proposed system approximates it as the instant in time where the distance between the two feet (i.e., width of the rectangular bounding box fitted onto the silhouettes) is maximum [23], as illustrated in Figure 2b.

**Figure 3:**
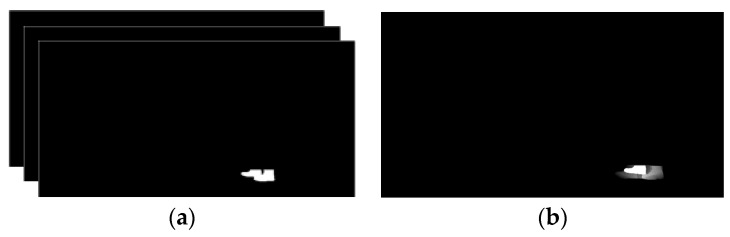

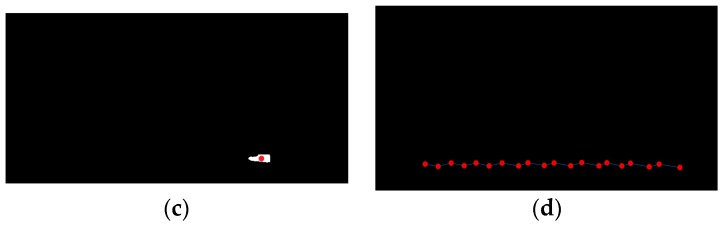
(**a**) Segmented feet silhouettes between two initial contacts; (**b**) AFI obtained by averaging the feet silhouettes; (**c**) position of the foot flat obtained by applying a threshold; (**d**) centroids of foot flat obtained for the entire video sequence.

Moreover, to obtain foot flat positions, only the feet region of the silhouettes is of interest, so an average feet image, AFI(x,y,t), can be created by keeping only the lower 10% fraction of the silhouettes, selected according to the human anatomy ratio [34]. The AFI is computed by averaging the resulting T feet silhouettes images, $I_{feet\;}\left(x,y,t\right)$, available between two initial contacts, according to Equation (1), and illustrated in Figure 3a,b. The averaging process makes the system more robust against any uncertainty in the estimation of the initial contact instant:

(1) $$AFI\left(x,y,t\right)=\frac{1}{T}\overset{T}{\underset{t=1}{\sum }}I_{feet}\left(x,y,t\right)$$

Since the AFI highlights the foot when it is in complete contact with the ground, by applying the Otsu thresholding [35] to the AFI the position of the foot flat can be obtained, as illustrated in Figure 3c. The resulting foot flat positions are used for computation of feet related features as detailed in the following subsections.

#### 2.2.1. Step Length (*SL*)

The step length can be measured using the foot flat positions obtained from the entire video sequence. However, due to the lack of depth information, there can be a significant difference between the scales of the two feet. Thus, to minimize errors, the proposed system computes the centroid of each foot flat and measures the Euclidean distance between two consecutive centroids as the step length.

Knowing the walking direction of an individual with respect to the camera, the proposed system can identify the foot closer to the camera as the right or the left foot. Thus, the proposed system can estimate both the left and right step lengths. To identify which foot is closer to the camera, since depth information is not available, the foot flat centroid positions can be used. As illustrated in Figure 3d, the centroid of the foot further away from the camera appears at a more elevated position in the image. Thus, by comparing the y-coordinate of the centroids, the step lengths can be classified as either left or the right step lengths. Since the video sequence contains multiple gait cycles and thus allow computing multiple feature values, a median is computed to increase the proposed system’s robustness to outliers [23]. So, the proposed system computes the median of the left and right step lengths as $SL_{i}^{left}$ and $SL_{j}^{right}$, respectively. A step length symmetry score, $SL_{symm}$, can then be computed as the absolute difference between the medians of left and the right step lengths, according to Equation (2). Since, the silhouettes are normalized during the pre-processing step the symmetry score remains consistent across different video sequences:

(2) $$\;SL_{symm\;}=\left|median\left(SL_{i}^{left}\right)-median\left(SL_{j}^{right}\right)\right|$$

#### 2.2.2. Normalized Step Count (*C*) and Speed (*S*)

The proposed system can also compute the normalized step count and speed of an individual’s movement using the foot flat information. Normalized step count, C, is computed as the total number of foot flat instances, k, divided by the total distance travelled, according to Equation (3). The distance travelled is measured as the length summation of the n observed steps, SL. The distance is measured in pixels due to the silhouette height normalization performed during pre-processing, which makes the system robust to scale changes:

(3) $$\;C=\frac{k}{\sum_{i=1\;}^{n}SL_{i}}$$

The speed, S, of an individual’s movement is computed by dividing the total distance travelled by the duration of the video sequence, according to Equation (4). The duration of the video sequence, d (in seconds), is measured between the first and last initial contacts:

(4) $$\;S=\frac{1}{d}\overset{n}{\underset{i=1\;}{\sum }}SL_{i}$$

#### 2.2.3. Foot Flat Ratio (*FFR*)

A walking video sequence is composed of several repetitions of a gait cycle, delimited by the initial contacts of the observed foot. It is also possible to divide each gait cycle into two phases separated by a “toe off” event, occurring when the toe of the foot being observed just leaves the ground. The phase before the toe off is called the stance phase, while the phase following the toe off is called the swing phase. Using the initial contact and the toe off, the proposed system can estimate the duration of the stance phase. As discussed in [23], the duration of the stance and swing phases are not unique enough to distinguish between different types of gait impairments. However, the amount of time the foot remains in complete contact with the ground, during the stance phase, can change significantly depending on the type of gait impairments. Thus, the proposed system computes a “foot flat ratio” feature, which can be defined as the fraction of the stance phase for which the foot remains in complete contact with the ground.

**Figure 4:**
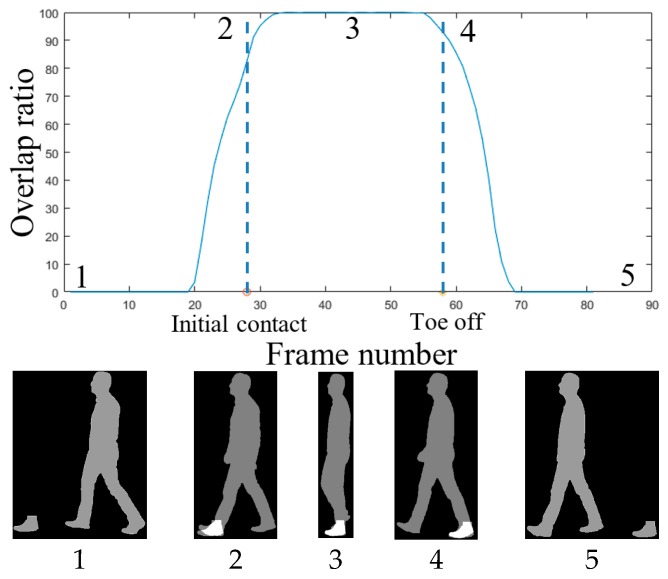
Plot representing the foot flat overlap ratio (**top**) and the corresponding silhouettes (**bottom**).

To compute the foot flat ratio, FFR, the proposed system measures the amount of overlap between the foot flat and the silhouettes belonging to the corresponding stance phase. It estimates the foot flat duration by counting the number of frames for which the foot flat is completely covered by the silhouettes—see Figure 4. Foot flat ratio values, for both the left and right feet, can then be computed according to Equation (5):

(5) $$\;FFR=\frac{flat\;foot\;duration\;}{stance\;phase\;duration\;}$$

### 2.3. Body Related Feature Extraction

Apart from the feet, the body of an individual can also provide significant information about gait impairments that an individual may be suffering from. For example, an individual’s movements can become severely restricted and the posture of the individual can be severely altered due to disorders such as Parkinson’s disease [2]. Thus, a measurement of the amount of movement and posture instability can be useful for classifying such gait impairments. Also, in some cases, the movement of a single limb may be restricted, or more restricted than the other limb. Therefore, analyzing the movement for every half gait cycle can be useful.

#### 2.3.1. Amount of Movement (*AOM*)

The proposed system computes the amount of movement during every half gait cycle using the entropy. However, unlike what was done for feet related features computation, here the half gait cycle is delimited by the mid-stance and mid-swing events, as it contains the part of the gait cycle where individuals shift the body weight from one side of the body to the other. The proposed system can thus capture movement restrictions while shifting weight onto the impaired side of the body. The mid-stance and mid-swing instants are approximated as the instants of the gait cycle when the distance between the two feet is minimum, corresponding to the valleys in the representation of Figure 1b. The silhouettes belonging to the considered half gait cycle, numbered from 1 to *P*, can then be cropped, $I_{c}(x,y,p$) and averaged to obtain the half cycle GEI, $GEI_{hc}\left(x,y,p\right)$, according to Equation (6) [17]:

(6) $$\;GEI_{hc\;}\left(x,y,p\right)=\frac{1}{P}\overset{P}{\underset{p=1}{\sum }}I_{c}\left(x,y,p\right)$$

**Figure 5:**
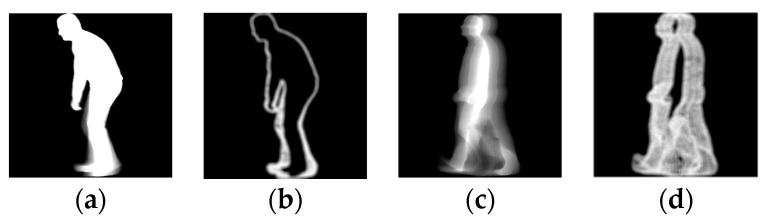
Half cycle GEI computed using impaired (**a**) and healthy (**c**) gait silhouettes, and the corresponding entropy representations (**b**,**d**).

The “amount of movement” feature, AOM, can then be computed over the half cycle GEI according to Equation (7), where $P_{i}$ is the probability that the difference between two adjacent pixels is equal to *i*. As illustrated in Figure 5b,d, the restriction in movement can be effectively represented using Shannon entropy [36]:

(7) $$\;AOM\;=-\underset{i}{\sum }P_{i}log_{2}P_{i}\;$$

Following the indexing of the foot flat, the amount of movement features can also be classified into left, $AOM_{i}^{left}$ and right, $AOM_{i}^{rigth}$, according to the foot that enters into an initial contact during the considered half gait cycle. A symmetry measure, $AOM_{symm}$, can also be computed to represent the difference in movement between left and right, according to Equation (8):

(8) $$\;AOM_{symm\;}=\left|median\left(AOM_{i}^{left}\right)-median\left(AOM_{j}^{right}\right)\right|$$

#### 2.3.2. Shift in COG (*COG_shift_*)

**Figure 6:**
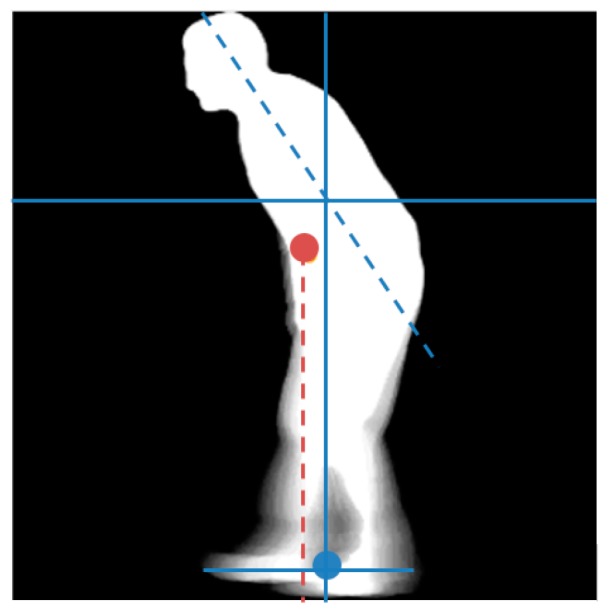
GEI highlighting shift in COG (middle silhouette point) with respect to the COS (lower silhouette point) and the orientation of the torso.

As illustrated in Figure 6, certain types of gait impairments caused by disorders such as Parkinson’s disease, can affect the posture of an individual, being reflected as a change in the orientation of the torso and therefore as a shift in the individual’s COG with respect to the COS (center of the base of support). Healthy individuals walk such that their COG and COS are always approximately vertically aligned. The proposed system computes the amount of shift using a GEI computed similarly to (6), but over the entire gait cycle. Using the GEI provides robustness to variations in the shift in COG occurring at different instants of the gait cycle. The COG is measured as a weighted centroid of the GEI, using the GEI intensity values as weights. The COS is measured as the center of the feet region of the GEI, obtained by segmenting the feet using a human anatomy ratio [34]. The shift in COG, $COG_{shift}$, can then be computed as the absolute difference between the horizontal coordinates of the COG and COS, according to Equation (9):

(9) $$\;COG_{shift\;}=\left|COG_{x}-COS_{x}\right|$$

#### 2.3.3. Torso Orientation (*TO*)

The last feature considered, called torso orientation, TO, is also computed using the complete GEI. The proposed system selects the torso using the human anatomy ratios presented in [34]. It then performs principal component analysis over the torso and measures the angle (*θ*) between the horizontal axis and the first principal component, PC($PC_{x}$, $\;PC_{y}$), according to Equation (10) [33]:

(10) $$\;TO\;\left(\theta^{\circ }\right)=\left|tan^{-1}\left(\frac{PC_{y}}{PC_{x}}\right)\times \frac{180}{\pi }\right|$$

### 2.4. Classification

The proposed system can perform classification in two different ways. First, the system can use each feature to classify gait as either normal or impaired. The paper tests the discriminative power of each feature to classify gait as normal or impaired by using the two-sample *t*-test with unequal variances [37]. This test can be used to determine whether two samples are drawn from the same or from different population groups. Thus, the results of the test can be interpreted to identify the features that are more significant in differentiating between normal and impaired gait.

Although an individual feature can be used to classify gait as either normal or impaired, the type of impairments that can be detected may vary depending on the features used. For instance, features such as shift in COG or torso orientation can help detect posture instabilities, features such as step length or amount of movement can be used to detect asymmetric gait, while features such as foot flat ratio, normalized step count or speed can be used to detect slow moving gait and other deviations. Thus, using all the available features together can allow the proposed system to further classify gait based on the type of impairment. It can also allow identification of disorders, or even determine the severity of such disorders. For example, Parkinson’s disease reduces the walking speed, alters the posture and restricts the movement of an individual, while disorders such as hemiplegia restrict the movement of a single side of the body.

The proposed system can distinguish between gait impairments caused by such disorders using the proposed biomechanical gait features. It is also possible to identify the side of the body whose movement is more affected, such as in the case of hemiplegia, as the considered features allow differentiating between left and right side impairments. The proposed system performs such classifications using a SVM, a discriminative classifier that separates data using a hyperplane [38]. To improve the classification accuracy of the proposed system, the SVM is used with a quadratic kernel.

### 2.5. Test Protocol

Two different tests are considered to evaluate the proposed system. The first test analyzes the discriminative power of each feature, and the second test analyzes the classification accuracy of the whole system. Thus, to successfully evaluate the proposed system, the following protocols are defined.

#### 2.5.1. Two Sample *t*-Test

To test the ability of each feature to differentiate between normal (NM) and impaired (FB, RL, LL) gait sequences, a two-sample *t*-test with unequal variances and a significance level of 0.05 is conducted. The null hypothesis for the test states that the two samples are drawn from the same population group. Thus, given two sample sets of a feature, drawn from the available NM and one of the three impaired sequence groups, the test will return a *p*-value, which can be used to either accept or reject the null hypothesis. The *p*-value is the probability of finding the observed, or more extreme, result when the null hypothesis of a test is true. If the *p*-value is above the significance level of the test (in this case 0.05), the null hypothesis is accepted. Otherwise, the alternate hypothesis, that the two sample sets are drawn from two different population groups, is accepted. Accepting the alternate hypothesis also suggests that the feature being tested is discriminative enough to differentiate between the two samples.

#### 2.5.2. Gait Type Classification Using SVM

The classification accuracy the proposed system is analyzed using a fivefold cross-validation technique. The technique divides the data into five sets, where each set contains features from two different Individuals. Thus, the training and testing set are mutually exclusive, with respect to the participating individuals. Next, the classification step is repeated 5 times such that each time, one of the five sets is used for testing and the other four sets are used for training the system. Finally, an average is computed to represent the classification accuracy of the system. The advantage of using fivefold cross-validation is that the variance of the resulting estimate is reduced as the results do not depend on a particular way of partitioning the data.

## 3. Results

The ability of the features to distinguish the left and right limbs, assess gait symmetry, posture instabilities, speed changes and other deviations from normal gait allows the proposed system to distinguish between different types of gait impairments. However, due to the lack of publicly available databases, the proposed system is currently tested only on the INIT gait database presented in [23]. This database contains binary silhouettes of ten individuals (nine males, one female) simulating eight different gait impairments. All the sequences are acquired in a lateral view and the type of impairment is manually annotated to create the ground truth. Each individual is recorded two different times in a LABCOM studio [23] at 30 fps, capturing multiple gait cycles per each recorded sequence. Since the first four simulated impairments correspond to restricted arm movement, testing is done considering the other four feet related impairment simulations, for which the proposed system can compute relevant feet and body related features. The tested sequences are labelled as: restricted full body movement (FB), restricted right leg movement (RL), restricted left leg movement (LL) and normal gait (NM). FB sequences simulate disorders such as Parkinson disease while, RL and LL sequences simulate disorders such as hemiplegia.

Following the protocol described in Section 2.5.1, each feature obtained from the proposed system is tested for its ability to characterize gait impairments. *t*-Test results are presented in Table 1, while the mean and the standard deviation of the features are presented in Table 2. The decision to perform the *t*-test is made considering the size of the INIT database presented in [23]. Assuming that feature samples are normally distributed, the *t*-test provides an assessment of the reliability of a given feature in in being able to differentiate between normal and impaired gait, while taking the sample size into consideration. Thus, the features resulting in lower *p*-values can be expected to be more significant in differentiating between normal and impaired gait.

The proposed system is further tested to check its ability to classify different gait impairments into different groups. The system is tested using a fivefold cross validation technique, following the protocol described in Section 2.5.2. Its classification accuracy is reported in Table 3, along with the confusion matrix in Table 4.

## 4. Discussion

The proposed system can identify the left and the right leg, which allows a more complete characterization of gait impairments than was possible with the system described in [23]. This can be concluded from Table 1, where each entry presents the *p*-value of the two-sample *t*-test. The first row of the table presents the results between the NM and FB groups. The results show significantly low *p*-values for almost all the computed features. The lowest values are observed for step length, amount of movement and torso orientation, suggesting that these features are more significant when differentiating between NM and FB gait. The low *p*-values are due to shorter step lengths, restricted body movement and a hunched posture. The hunched posture also causes a significant difference in the shift in the COG feature, represented by a low *p*-value in Table 1. The speed of the individuals in the NM group is also significantly different from the FB group. This is represented by low *p*-values for the speed, normalized step count and the foot flat ratio. The only feature that accepts the null hypothesis is the step length symmetry, indicating that when differentiating between NM and FB gait the symmetry feature will perform poorly, as in these cases the steps length of both the legs are similar. However, the step length symmetry and amount of movement symmetry features are significant when differentiating between NM and RL/LL gait, as indicated by their low *p*-values in Table 1. The *p*-value for the other features, such as step length, foot flat ratio, speed, normalized step count and shift in COG, are also low suggesting that they are significant enough to distinguish between the two groups. The features that accept the null hypothesis for RL/LL gait are the torso orientation and the amount of movement for the unrestricted side of the body. However, this is expected as the torso orientation feature is only effective in severe posture instability cases, such as hunchbacks, and the amount of movement of the unrestricted side is expected to be similar to NM group.

To better illustrate the difference between the different types of gait, each entry in Table 2 presents the mean and standard deviation of each feature belonging to the respective group. Using Table 2, it can be seen that the FB gait is significantly slower than the NM gait, indicated by low speed, high normalized step count and a large fraction of time spent in foot flat during stance phase. The step lengths are also significantly shorter than the NM group. However, there is no significant difference between the left and the right foot, as indicated by low step length symmetry values and the amount of movement symmetry values. Also, the bending of torso and the shift in COG is significantly larger than the NM gait—see Table 2. For the RL/LL groups, the restricted leg/side of the body is indicated by short step length and low amount of movement (entropy values) in Table 2. However, the gait is relatively fast as indicated by the higher speed and lower normalized step count values. Finally, it should be noted that the shift in COG feature is effective in differentiating between normal and all the three types of impaired gait, as illustrated in Table 1, but it is not very precise in its measurement—see Table 2. The low precision in the measurement is caused by camera distortions, whose effect is severe, especially at the start and at the end of the gait sequence. Although its precision is not as good as that of other features, its ability to differentiate between different gait impairments allows the proposed system to successfully classify the gait as being normal or impaired.

Next, the ability of the proposed system to classify gait across different impairment groups is tested following the fivefold cross-validation technique and the resulting classification accuracy is reported in Table 3. These results indicate that the proposed system performs extremely well, being able to classify gait sequences as FB, RL, LL or NM with a correct classification accuracy of 98.8%. The results also indicate that there is a significant variation between the feature values observed for each group. Table 3 also contains a comparison of the proposed system against the state-of-the-art markerless 2D video based systems, tested using the same fivefold cross-validation technique. The leg angle method, presented in [24], can be effective when there is sufficient separation between legs. However, even in NM group, it becomes difficult to distinguish between the two legs during mid stance and mid swing phases, while in the FB sequences there is no separation between the two legs during the entire gait cycle. The work presented in [22] uses a GEI along with SVM to perform classification of gait impairments. The use of GEI allows the method to successfully differentiate between FB and NM groups, but there are significant misclassifications between the NM, RL and LL groups, reducing the overall classification accuracy to 75%—see Table 3. Even with a linear SVM, the proposed system performs better than the GEI method [22] with a correct classification accuracy of 95.0%, following the fivefold cross-validation technique. A second drawback of the GEI method [22] is that the GEIs used for the classification process do not provide any additional information about the gait impairments, while the proposed system provides measurable features that can be used to further analyze an individual’s gait. Thus, it can be concluded that the biomechanical features used by the proposed system provide a better representation for gait impairment detection and classification. The performance of the proposed system can also be analyzed using the confusion matrix presented in Table 4. It shows that the proposed system performs extremely well—in fact, only a single sequence is misclassified which, due to the limited size of the available database, results in a 5% penalty to the classification accuracy of the RL group. It should also be noted that the falsely classified sequence is poorly simulated, as can be observed by the mean step length of the left and right legs, leading to its classification into the NM group. The results from the Table 4 show that the proposed system operates with an average recall of 98.75% and precision of 98.80%. The average recall and precision of the methods presented in [22] is 75% and 76.07%, and [24] is 72.5% and 72.89% respectively, suggesting that the proposed system performs significantly better than the state-of-the-art methods. Also, the goodness index of the proposed system is 0.0177 which makes it “optimal” according to [39].

## 5. Conclusions

The paper presents a novel markerless system that performs successful acquisition and evaluation of an individual’s gait using a single 2D video camera. It evaluates individuals’ gait using biomechanical gait features acquired from their binary silhouettes. These features allow the proposed system to classify an individual’s gait across different gait impairments. The classification accuracy of the proposed system is significantly better than the current state-of-the-art.

The features acquired by the proposed system can be classified into two types. The first type is related to the feet of an individual. They include features such as step length, normalized step count, speed and the fraction of foot flat during a stance phase. The proposed system can distinguish among the features obtained from the left and the right foot, thus allowing the system to estimate gait symmetry. The second type of features are related to the entire body of an individual. They include features such as the amount of movement while walking, torso orientation and the shift in the COG with respect to its COS. Similarly, to feet related features, features such as the amount of movement are computed separately for either feet, to compute a symmetry score. Apart from detecting the left-right symmetry, the proposed system also detects posture instabilities using torso orientation and the shift in COG, while other features such as normalized step count and speed are used to detect the deviation from the normal gait. Using a SVM classifier, the proposed system performs almost 100% correct classification across four different types of gait on the INIT database.

Due to the lack of publicly available databases, the proposed system is currently tested on a database containing only twenty simulated sequences for each impairment type. Therefore, the future work will consider testing of the proposed system on a larger database, containing a larger variation in gait impairments, acquired from real patients. The resulting features will also be validated using the gold standard sensor or vision-based systems. The low precision of the shift in COG feature will be improved by rectifying the camera distortions in the pre-processing step. Another possible extension is to include features reflecting different arm related impairments, thus allowing the proposed system to perform an improved evaluation of an individual’s health. The features can also be explored to predict fall risks in elderly populations.

## Figures and Tables

**Table 1:** Two sample *t*-test with unequal variances and significance level of 0.05 performed between normal and impaired gait.

	FB	RL	LL
SL Left	1.56 × 10^−21^	2.59 × 10^−2^	4.01 × 10^−9^
SL Right	1.05 × 10^−23^	1.44 × 10^−10^	3.88 × 10^−2^
SL Symmetry	1.74 × 10^−1^	5.29 × 10^−10^	1.38 × 10^−9^
FFR Left	4.48 × 10^−7^	1.00 × 10^−3^	4.89 × 10^−4^
FFR Right	4.82 × 10^−7^	3.25 × 10^−4^	1.08 × 10^−1^
*S*	7.02 × 10^−16^	1.33 × 10^−4^	1.48 × 10^−4^
*C*	4.47 × 10^−11^	2.50 × 10^−5^	1.11 × 10^−5^
*TO*	2.94 × 10^−14^	6.46 × 10^−1^	8.75 × 10^−1^
*COG_shift_*	6.87 × 10^−3^	1.54 × 10^−4^	4.97 × 10^−2^
AOM Left	8.04 × 10^−16^	3.328 × 10^−1^	2.04 × 10^−9^
AOM Right	1.98 × 10^−19^	1.25 × 10^−7^	5.97 × 10^−1^
AOM Symmetry	2.46 × 10^−3^	1.21 × 10^−7^	1.94 × 10^−9^

**Table 2:** Mean and standard deviation of all the observed gait features belonging to different groups.

	FB	RL	LL	NM
SL Left (pixels)	48.49 ± 12.70	115.16 ± 13.24	70.95 ± 25.26	124.19 ± 11.28
SL Right (pixels)	41.19 ± 10.24	63.24 ± 23.36	108.69 ± 21.90	120.68 ± 11.56
SL Symmetry (pixels)	7.28 ± 6.18	51.92 ± 18.61	45.90 ± 17.20	5.137 ± 3.08
FFR Left (%)	0.80 ± 0.09	0.70 ± 0.05	0.68 ± 0.09	0.64 ± 0.04
FFR Right (%)	0.75 ± 0.09	0.66 ± 0.05	0.67 ± 0.06	0.60 ± 0.06
*S* (pixels/s)	37.04 ± 8.86	63.11 ± 15.31	64.77 ± 13.44	81.41 ± 11.37
*C* (steps/pixels)	0.025 ± 0.005	0.013 ± 0.002	0.013 ± 0.001	0.010 ± 0.000
*TO* (°)	62.87 ± 6.25	84.97 ± 3.00	85.25 ± 3.24	85.40 ± 2.85
*COG_shift_* (pixels)	12.39 ± 5.93	4.65 ± 2.25	6.40 ± 2.85	8.08 ± 2.84
AOM Left (entropy)	1.76 ± 0.31	3.12 ± 0.14	2.51 ± 0.29	3.17 ± 0.11
AOM Right (entropy)	1.58 ± 0.25	2.35 ± 0.42	3.01 ± 0.24	3.10 ± 0.11
AOM Symmetry (entropy)	0.17 ± 0.12	0.77 ± 0.39	0.55 ± 0.21	0.06 ± 0.05

**Table 3:** Classification accuracy of the proposed and state-of-the-art systems.

Method	Classification Accuracy
Leg angle method [24]	72.5%
GEI method [22]	75.0%
Proposed system	98.8%

**Table 4:** Confusion matrix for the proposed system.

	Predicted Group				
		FB	RL	LL	NM
True Group	FB	100%	0%	0%	0%
	RL	0%	95%	0%	5%
	LL	0%	0%	100%	0%
	NM	0%	0%	0%	100%
